# Setting process control chart limits for rounded-off measurements

**DOI:** 10.1016/j.heliyon.2023.e13655

**Published:** 2023-02-21

**Authors:** Ran Etgar, Sarit Freund

**Affiliations:** Department of Industrial Engineering, Faculty of Engineering, Ruppin Academic Centre, Emek-Hefer, Israel

**Keywords:** Quality control, Statistical process control, Round-off, Measurement, Rounding error

## Abstract

Measurements can often be imprecise and subjected to rounding-off. Typically, this rounding-off is ignored and assumed to have little to no effect. However, when the measuring scale step is not negligible, it may affect statistical control tools such as $\bar{X}$-chart. Designing statistical process controls without considering the effects of rounding leads to high exposure to false negative results. This study illustrates the effects of rounding on the X-chart and shows that the result may further deteriorate due to asymmetry (incompatibility of the process and the measuring device parameters). A new simple method to design control limits is proposed, based on maintaining the original characteristics of the chart as devised by Shewhart.

## 1 Introduction

Rounding off is a common and necessary practice. When dealing with continuous data, there is a built-in limit to the ability to measure the observed quantity accurately. The implicit assumption in most standard statistical methods is that the acquired (i.e. measured) data are identical to the actual data, since the measurements are “exact”. Typically, this implicit assumption is justified and has no effect on the result (and therefore on the analyses), since the rounding-off is minor. However, in some cases where the rounding-off is large, this assumption may lead to biases and incorrect statistical analyses [1].

Measurement is a fundamental activity in all the sciences and the ability to measure is a prerequisite to data collection in any statistical study. If a measured variable is real-valued, it is only observed to the nearest kth decimal floating point [2]. The difference between the measured means and the continuous version is small but can accumulate substantially over batches of measurements [3]. For example, in one study, the estimation of a parameter μ was investigated [4]. The findings showed that in many industrial applications where digital instruments are used, data collection often involves relatively crude gaging. Because the industry 4.0 (r)evolution relies heavily on such sensors [[5], [6], [7]], the need to deal with such errors has increased exponentially. Although it is tempting to treat the rounding error as another kind of measurement error, there is an intrinsic difference between the two, since the rounding error depends on the actual value whereas the measurement error does not [8,9].

Digital measuring equipment typically has a discrete scale with distinct steps (h). This step is the result of the t level of quantization of the equipment (e.g., a thermometer that can measure in steps of $0.1^{0}C$). Analog equipment is also subjected to a similar bias, because the results are rounded off to the nearest scale mark. However, even when the measuring device can produce highly accurate results, the storage systems may round them off [10]. Ignoring rounding-off is warranted when the standard deviation (σ) of the measured parameter is significantly smaller than the value of h. A rule of thumb is that the value of σ should be at least twice the value of h [11]. The higher the ratio of σh (denoted by δ) the less ignoring the rounding-off is justified. An example is depicted in Section 6.

### 1.1 A brief glance at the literature on rounding-off

The need for rounding-off and its effect on the results is obviously not a new phenomenon. As early as 1898 Sheppard [12] pointed this out and in 1957 [13] a rule of thumb was suggested to decide when ignoring the issue is reasonable. Dempster and Rubin [14] examined the effect of rounding on the linear regression model (ignoring the rounding-off effect, using Sheppard's correction, and correcting the covariance matrix). More recent extensions [15,16] have confirmed that the effects of round-off (or “grouping”) are slight when the interval width (h) is small. In 1996 [17] the subject of grouped measurements was examined and its expensive or impossible measurements, where classifying units in groups is economical and presented control chart techniques for grouped data. The article demonstrated that using the midpoint for each group value as the rounded data can harm the results of the control charts that are designed for use with exact measurement. In the following year [18] the effect of rounded data on R charts was examined and conclusions about data rounding, without adversely affecting the control limit factors on this type of charts were achieved. In the same year Bryce et al. [19] assessed the quality of four estimators of standard deviation – sample standard deviation, mean moving range, median moving range and Ciminera-Tukey measure. They used computer simulations to evaluate the four estimators control limits for monitoring individual observations and to detect special causes. The effect of rounded data on R-chart was demonstrated [20] in which the term degree of precision as r = w/σ was defined (w is the width of the rounded interval of x, and σ is the standard deviation of x). Rules concerning the degree of precision that should be used when recording data were suggested. In 2001 McNames et al. Examined the influence of mean Quantization on control chart Q [21]. The influence of rounded data on the variance, regression parameters and the distortion in comparison to unrounded data was explored [22] and expression to evaluate this distortion depending on the length of the rounding interval was suggested.

In 2010 Meneces et al. [23] examined the effect of exponentially weighted moving average control charts using Monte Carlo approach and presented the detection capabilities (as a function of measurement resolution.

The general case is that both the variance and the mean need to be found, but in some cases, the mean can be assumed to be known from the production process setting [24]. The variance, on the other hand, needs to be estimated from the measures. Since the straightforward calculation (i.e. ignoring the rounding-off) is inadequate, several attempts have been suggested to find the variance. Sheppard [12] proposed a correction method to deal with this problem, which was later criticized [1] by showing that in some cases it failed to solve the problem. Based on work by Schader and Shmid [25], Gertsbakh [11] put forward a maximum-likelihood (ML) based method to estimate both the mean and the standard deviation. In 2012, Benson-Karhi et al. [26] suggested a method for calculating the variance from the data using the method of moments (MoM) when the mean is known, and showed it to be superior to the ML based method. Lee and Vardeman [27] expanded on a previous work [28] and discussed the case of estimating the variance (for normally distributed processes) when neither the mean nor the variance is known. They used a parametric likelihood-based method, the Maximum Likelihood Estimator, which they compared to the “classic” method (i.e. ignoring the rounding-off effect). They also [29] demonstrated how it can be used to produce reliable confidence procedures for the two variance components. Benson-Karhi et al. [30] improved their previous technique by combining the MoM with a calibration technique. By using MSE as a criterion and a simulation to generate a database, they showed that their technique outperformed the “classic” method, Sheppard's correction estimator, and the Vardeman and Schader approach [1,25]. Since most studies have focused on estimating the mean and variance for the case of normally distributed parameters, it is worth inquiring whether this is justifiable. Box, Luceno [31] defended this approach by pointing out that the random error averages the number of component errors, so that the central limit theorem is applicable and random errors tend to have a normal rather than another distribution.

Despite the fact that there are several approaches to finding the mean and variance for rounded-off measurements, no similar attempts have been made to examine the effect of rounding-off on control charts [26]. Ever since Shewhart [32] presented the concept of control charts, they have been the most popular method for maintaining statistical process control [33,34]. The most well-known and widely used is the $\bar{X}$-chart [35] for monitoring the mean.

The Shewhart $\bar{X}$-chart is a set of two control limits: the upper control limit (UCL) and the lower control limit (LCL) [36]. These limits are calculated symmetrically around the mean, as depicted in Eq. (1):

$$UCL=\mu +k\frac{\sigma }{\sqrt{n}}$$

(1) $$LCL=\mu -k\frac{\sigma }{\sqrt{n}}$$

where μ is the mean, k is a parameter set to the rate of false alarms. Typically, a value of 3 is chosen so that the rate of false alarms is 0.0027 (since Φ(−3)=1−Φ(3) = 0.00135). When a sample of size n is taken and its average value ($\bar{X}$) either goes above the UCL or below the LCL, an alarm is set to denote that the process is likely out of control. An in-depth literature review of the $\bar{X}$-chart can be found in Ref. [37]. Crucially, however, these control limits do not take the rounding-off of the measured variable into consideration. Therefore, the design of the UCL and LCL may lead to unwanted false alarm values. Furthermore, the symmetrical nature of the UCL/LCL around the mean may prove undesirable in the context of rough round-offs. To respond to these needs, this paper suggests a new method to set the control limits.

The remainder of this paper is organized as follows. In the second section, the distribution of the expected results is described and the distribution function of the measured control variable is set. The third section outlines the problems caused by ignoring the rounding of the measurements and by using “classical” statistical process control. The fourth section delves into the effect of asymmetry and the fifth provides a detailed method to set the control limits. An example of a real-life case is depicted in Section 6. The final section provides a summary and draws key conclusions.

## 2 Measurement distribution

When measuring a value X (the ‘measurand’ [26,30]) the observed value (Y) is rounded off. The relationship between these two values is expressed in Eq. (2).

(2) Y=X+ε

where ε is the rounded-off error. Since X is assumed to have normal distribution ($X∼N(\mu ,\sigma^{2}$)), the probability distribution function (pdf) of Y is depicted in Eq. (3).

(3) $$P\left(Y=y\right)=\Phi \left(\frac{yh+\frac{h}{2}-\mu }{\sigma }\right)-\Phi \left(\frac{yh-\frac{h}{2}-\mu }{\sigma }\right)\;for\;any\;integer\;y$$

where X is a continuous variable, Y is discrete and therefore is limited to specific values.

To avoid having to work with different units for the random variables (the mean and σ), for the remainder of this paper it is assumed (without loss of generality) that σ=1 (i.e., all values are measured in units of σ). Therefore (Eq. (3)) is replaced by Eq. (4).

(4) P(Y=y)=Φ((y+0.5)h−μ)−Φ((y−0.5)−μ)forintegery

where both h and μ are measured in units of σ.

**Fig. 1:**
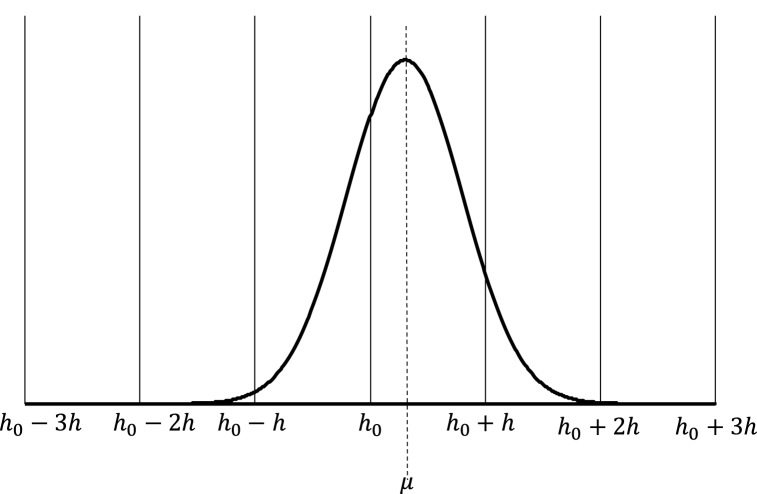
Relationship between the X probabilty distribution function and the Y probability function.

Theoretically Y has infinite possible values (for y∈{−∞,…,−1,0,1,…∞}); however, Gertsbach [11] and Benson-Karhi [26] observed that five values of Y were enough when rounding is *rough*. For rough rounding, the value of Y is expressed in Eq. (5).

(5) $$Y\in \left\{h_{0}-2h,h_{0}-h,h_{0},h_{0}+h,h_{0}+2h\right\}$$

where $h_{0}$ is the mode, as depicted in Fig. 1.

This omission is justified by Gertsbach for rough values of δ (where δ is defined as $\frac{1}{h}$) where that 5 cases cause other values of Y to have negligible probability. For instance, with $\delta =\frac{1}{2}$ (i.e. the upper bound of ‘special’ measurement scheme) and for $\mu =h_{0}+\frac{h}{2}$, we obtain $P\left(Y=h_{0}+3h\right)=3.2\times 10^{-5}$.

Despite Gertsbach's and Benson's decision to omit, in this paper it is assumed that values of Y were set to 7 cases, as depicted in Eq. (6).

(6) $$Y\in \left\{h_{0}-3h,h_{0}-2h,h_{0}-h,h_{0},h_{0}+h,h_{0}+2h,h_{0}+3h\right\}$$

**Fig. 2:**
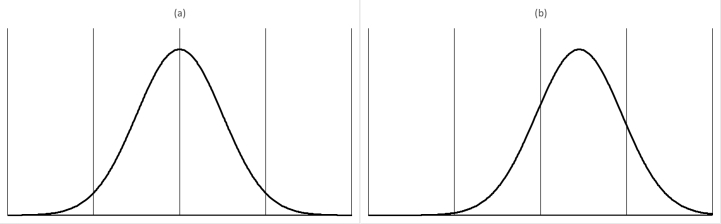
Relationship between X and Y: (a) Centered case. (b) Deviation from the center.
• Adding the two new values makes it possible to cope with less rough cases of rounding-off.

The rationale is as follows:

• Gertsbach's calculations are based on the implicit assumption that the process is centered (as depicted in Fig. 2(a)) or that the deviation from the center is minimal. Extreme deviations (i.e., of nearly 0.5 as depicted in Fig. 2(b)) cause the probability of being outside the set described in (Eq. (4)) to increase by more than one order of magnitude (see Section 4 for details).

As described in Section 1, the conditions for an alarm are:

(7) $$\bar{X}\leq LCL\;or\;\bar{X}\geq UCL$$

However, as X itself cannot be measured, $\bar{X}$ cannot be calculated, so we need to replace the condition in Eq. (7) with a condition for $\bar{Y}$.

From (Eq. (4)) it can be derived that the values of $\bar{Y}$ are limited to the ones described in Eq. (8).

(8) $$\bar{Y}\in \left\{h_{0}-3h,h_{0}-\left(3-\frac{1}{n}\right)h,h_{0}-\left(3-\frac{2}{n}\right)h,\ldots ,h_{0},h_{0}+\frac{h}{n},{\ldots ,h}_{0}+3h\right\}$$

To calculate the distribution function of $\bar{Y}$ let us define an auxiliary set of variables:

$n_{0}$ – number of ${y_{i}}^{\prime }$s equal $h_{0}$. $n_{-1}$ – number of ${y_{i}}^{\prime }$s equal $h_{0}-h$. $n_{-2}$ – number of ${y_{i}}^{\prime }$s equal $h_{0}-2h$. $n_{-3}$ – number of ${y_{i}}^{\prime }$ s equal $h_{0}-3h$. $n_{1}$ – number of ${y_{i}}^{\prime }$ s equal $h_{0}+h$. $n_{2}$ – number of ${y_{i}}^{\prime }$s equal $h_{0}+2h$. $n_{3}$ – number of ${y_{i}}^{\prime }$s equal $h_{0}+3h$.

Thus obviously the total number is as depicted in Eq. (9).

(9) $$n=\sum_{i=-3}^{3}n_{i}$$

The probability distribution function of $\bar{Y}$ can therefore be calculated (Eq. (10)).

(10) $$P\left(\bar{Y}=v\right)=P\left(\sum_{i=-3}^{3}i\cdot n_{i}=nv|\sum_{i=-3}^{3}n_{i}=n\right)$$

and from the multinomial distribution (Eq. (11)).

(11) $$P\left(\bar{Y}=v\right)=\left[\sum_{i=-3}^{3}\frac{n!}{\prod_{i=-3}^{3}n_{i}!}\prod_{i=-3}^{3}p_{i}^{n_{i}}|\sum_{i=-3}^{3}i\cdot n_{i}=nv,\sum_{i=-3}^{3}n_{i}=n\right]$$

where $p_{i}=P\left(Y=h_{0}+ih\right)$ (An example of this calculation for a small sample size of n=3 is described in Appendix 1).

## 3 False alarms caused by ignoring rough rounding

Setting the control limits (Eq. (1)) without considering the effects of rounding the measurement can lead to unwanted results. The discrete distribution of $\bar{Y}$ can lead to different results than the ones intended by the original design of these limits.

**Fig. 3:**
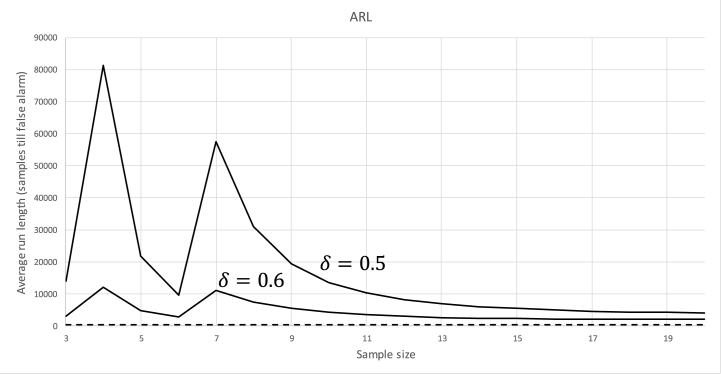
ARL for rounded samples. The dotted line indicates the “classical” ARL (shift from center = 0.4).

**Fig. 4:**
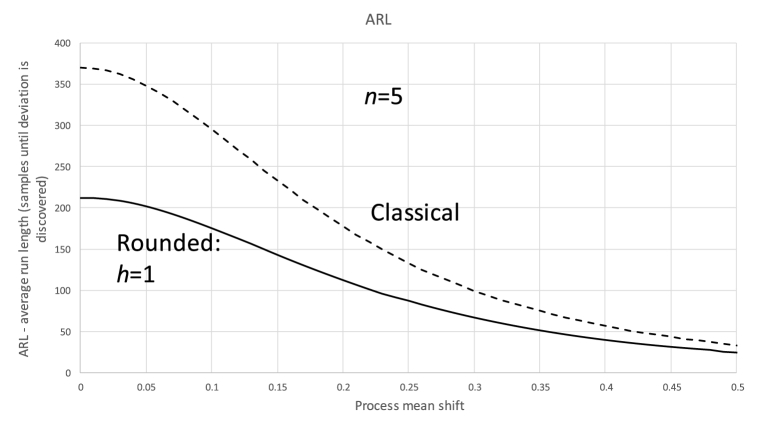
ARL for the case of sample size = 5 and h = 1.

To see this effect, let us assume a common case where the control limit parameter (k) is set to 3 (the value suggested by Shewhart [32]). For this case, the probability of a false alarm is Φ(−3)+(1−Φ(3))=0.0027. This value translates into an average run length (ARL) of 370 samples before there is a false alarm. As $\bar{Y}$ has discrete probability (Eq. (11)), this calculation is no longer valid. The actual probability of false alarms if the original control limits are maintained is depicted in Fig. 3. In this figure, it is demonstrated for two values of δ, that the classical limits are far from an adequate approximation of the ones we need. A decrease in the false alarm rate can be interpreted as a positive outcome, but the downside is of course the false positive rates (and, hence, the ARL until an alarm is raised). Fig. 4 compares the ARL of the non-rounded (classical) case to a case where h=1 (both with a sample size of 5). As expected the $\bar{X}$-chart's ability to detect shifts in the process mean drops sharply.

Fig. 3, Fig. 4 make it clear that to maintain the requirements of the $\bar{X}$-chart, a new method to establish the control limits is required.

## 4 Effect of asymmetry

**Fig. 5:**
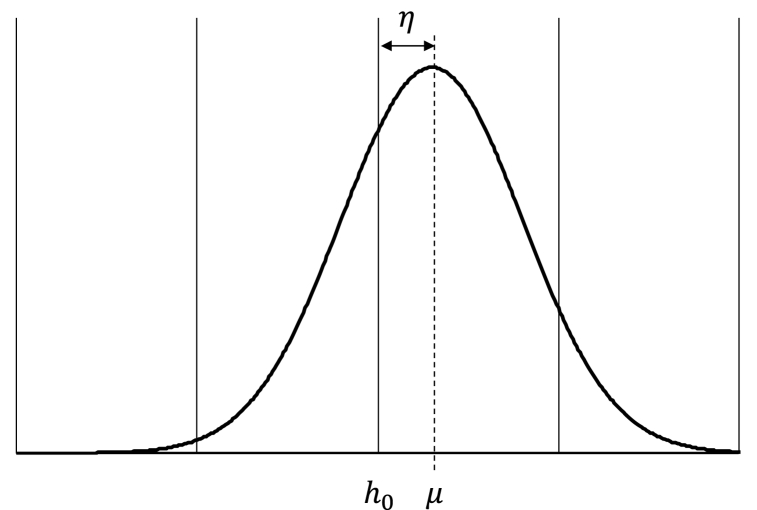
Asymmetry measure.

The classical $\bar{X}$-chart limits (Eq. (1)) are typically set symmetrically around the mean, due to the symmetrical nature of the normal curve. However, as depicted in Fig. 2(b), this symmetry is not preserved when the measurements are rounded since the measurement device is typically set to arbitrary human-related scale steps (e.g. rounded to a whole number of degrees when measuring temperature) whereas the actual mean is not constrained by this limitation (e.g. it can be 103.4 °C). This asymmetry (η) can be expressed as the deviation of the mean from the mode (in units of σ), as depicted in Fig. 5.

**Fig. 6:**
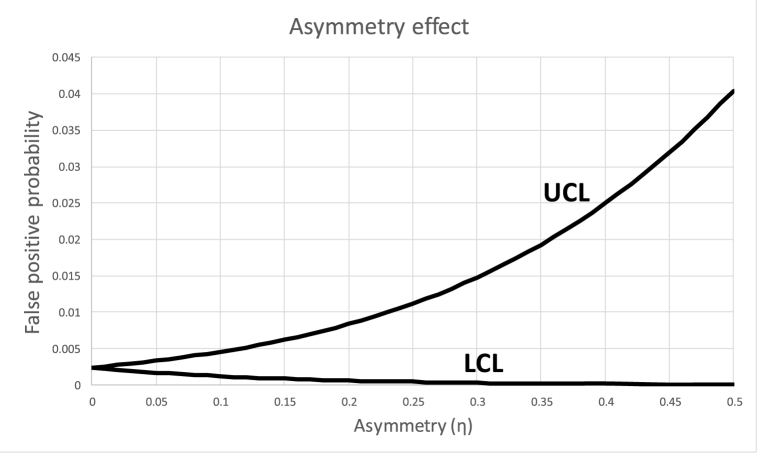
Probabilities of getting false positive results from crossing UCL and LCL.

Fig. 5 shows that −0.5h≤η≤0.5h, since larger (or smaller) asymmetry values will cause the mode to shift to the next step. Fig. 6 depicts the probabilities of crossing the UCL and the LCL for a sample size of 5 and h=1 as a function of the asymmetry.

The conclusion that derives from this section is that when setting the control limits, the asymmetry should be taken into account.

## 5 Setting control limits

Although there are various reasons to choose different values for parameter k (Eq. (1)), the most widely used value is 3 [[38], [39], [40], [41], [42]]. This value leads to an ARL (false positive) of about 370. This value is also divided equally between the UCL and the LCL (i.e., both have an ARL value of about 741).

It is worth mentioning the assumption that when setting the control limits the underling assumption is that the values of the mean and variance are known. Either by using special equipment (not available during the control phase) or by using the methods described in Section 1.

The optimal approach is to stick to the “conventional” ARL goals, and set the UCL and LCL accordingly. As demonstrated in the previous sections, the control limits should be calculated for various values of the sample size (n), the asymmetry (η) and the σ to h ratio (δ). To provide a practical and useable way to determine the limits, we need a simple tool which has approximately the required ARL value.

Unlike $\bar{X}$ which is continuous, $\bar{Y}$ is discrete and therefore (Eq. (7)) changes to Eq. (12).

(12) $$\bar{Y}<LCL\;or\;\bar{Y}>UCL$$

(Note that when the measured $\bar{Y}$ is *exactly* equal to one of the limits, there should not be an alarm).

Given the discrete nature of $\bar{Y}$ (Eq. (8)), it is possible to calculate the control limit values. The method developed in Eq. (11) and the demonstration depicted in Appendix 1 provide a simple way to calculate these limits. For each set of n,η and δ, the value UCL is calculated as the lowest value of $\bar{Y}$ for which the probability is calculated in Eq. (13).

(13) $$P\left(\bar{Y}>UCL\right)\geq 0.00135$$

Similarly, the LCL is the highest value of $\bar{Y}$ for which the probability is calculated in Eq. (14).

(14) $$P\left(\bar{Y}<LCL\right)\geq 0.00135$$

An example of the calculation of UCL and LCL is presented in Appendix 2.

**Fig. 7:**
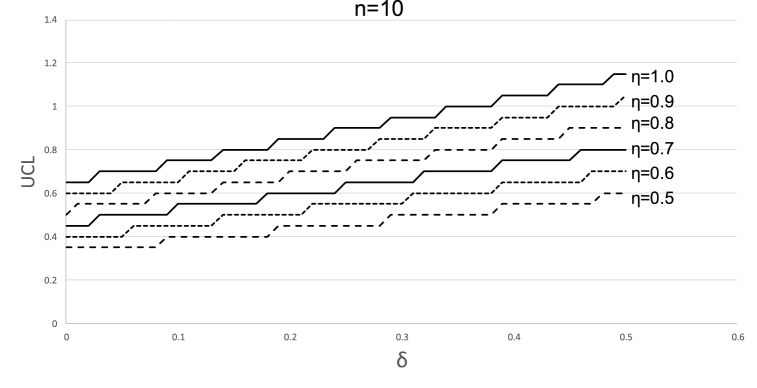
UCL for different combinations of η and δ. Sample size = 10.

However, it is impractical to provide a set of limits for each combination of n,η and δ. To construct a handy tool, let us examine the value of UCL as a function of η and δ (for a given n) as depicted in Fig. 7.

**Fig. 8:**
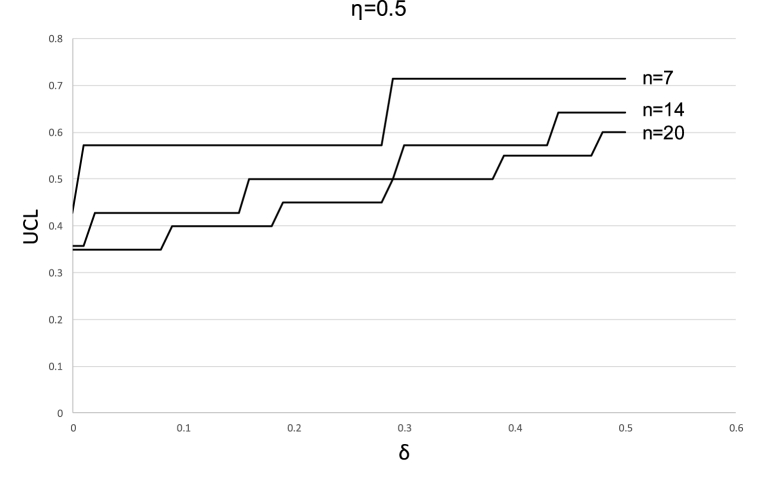
UCL for different combinations of sample sizes and δ. Asymmetry level = 0.5.

Fig. 7 clearly depicts (as known from (8)) that the value of the UCL does not change continuously with the change in δ, but rather increases by steps of $\frac{1}{n}$ for specific values of δ. This can also be seen in Fig. 8 which depicts the UCL from a different perspective (i.e., for a given value of asymmetry and various sample sizes and values of δ).

The discrete nature of the functions UCL(n,η,δ) and LCL(n,η,δ) can be turned into a simple table that designers can use to set limits for statistical process control. The designer does not need to go to the trouble of performing difficult calculations and can simply use pre-prepared values for most practical purposes. Appendix 3 provides the detailed procedure (and tables) for the practical implementation of setting the control limits.

## 6 SPC example

The following case demonstrates the problem that may arise from using rounded data with “regular” chart UCL and LCL.

High-end tomato seeds are very expensive; thus, the customer is promised to have the exact variety, germination duration and germination percentage (typically approaching 97%–100%). To achieve high germination percentage, the seeds go through a ‘priming’ process that increases the aforementioned percentage (the specific process is beyond the scope of this article and is typically a business secret of the seed company). After the process, the seeds go through various tests (aimed to ensure that the priming process did not decrease their shelf-life too much). Some of the tests are quite simple (e.g. germination in controlled environment for several days until sprouting), but some include more advanced measurements. One test is the measuring the content of the element phosphor (P). The content should average 20 μg with standard deviation of 1.5 μg (this is the original content and the purpose of the test is to ensure that it remained so). 8 seeds are taken from each batch. Although there exist (expensive) high accuracy devices that can measure phosphor content more accurately, the commercial measuring device can only measure with 1 μg accuracy.

Using ‘regular’ standards, the UCL and LCL are:

$$UCL=20+3\frac{1.5}{\sqrt{8}}=21.591$$

$$LCL=20-3\frac{1.5}{\sqrt{8}}=19.470$$

These values, if used, may lead to both false positive and false negative results. Table 1 depicts an example of false-negative event (i.e. the actual value was below the LCL, but the measured data did not spot this event). Table 2 depicts a false-positive event (where the exact data should not have caused an ‘alarm’ but the rounded date caused one anyhow).

**Table 1:** QC results (false-negative) - μgP.

Seed # Value	1	2	3	4	5	6	7	8	$\bar{X}$
Exact	20.561	19.11	19.845	19.586	18.731	18.148	20.089	19.453	19.440
Measured	21	19	20	20	19	18	20	19	19.500

**Table 2:** QC results (false-positive) - μgP.

Seed # Value	1	2	3	4	5	6	7	8	$\bar{X}$
Exact	18.475	20.34	20.294	20.407	19.168	19.713	18.576	19.367	19.543
Measured	18	20	20	20	19	20	19	19	19.375

## 7 Conclusion and future research

Rounding-off is the case in all continuous variable measurements. Generally, rounding has negligible effects, but occasionally it may be rougher than usual and produce unwanted effects on the statistical process control using $\bar{X}$-chart. When applied naïvely, the control chart may lead to erroneous decisions. In this paper, the drawbacks of using the classical control limits were investigated, including the effect of asymmetry on the results. A method to construct the distribution function of the measured parameter ($\bar{Y}$) was presented and used to generate a new way to construct the upper and lower control limits. The proposed method, although slightly more complicated than the original one suggested by Shewhart, is still convenient, accurate and only involves the use of two simple tables.

Although this research focused solely on the way to conduct $\bar{X}$-charts, the need also exists for other control charts (e.g. to control the variance, such as the R-chart) or if more robust charts [43] are needed, to six-sigma.

An interesting and valuable research is to examine the impact of rough measurements on gage repeatability and reproducibility (Gage R&R) – how to verify precision when the measured values are rounded?

Finally, this whole method is based on a rather controversial assumption – of normality. This assumption may not be correct in many cases, and nonlinear transformation to achieve normality may not be correct [44].

## Author contribution statement

Ran Etgar: Conceived and designed the experiments, Analyzed and interpreted the data, Wrote the paper.

Sarit Freund: Analyzed and interpreted the data, Wrote the paper.

## Funding statement

This research did not receive any specific grant from funding agencies in the public, commercial, or not-for-profit sectors.

## Data availability statement

Data included in article/supp. material/referenced in article.

## Declaration of interest's statement

The authors declare no competing interests.
