# Supplementary material for: Setting process control chart limits for rounded-off measurements
Source: Heliyon. 2023 Feb 21;9(3):e13655. doi: 10.1016/j.heliyon.2023.e13655 (PMC9982614; doi:10.1016/j.heliyon.2023.e13655)
Supplement: Multimedia component 1 [file mmc1.docx]

Appendix 1 – Calculating the $\overline{Y}$distribution

Calculating the $\overline{Y}$ is very straightforward, since the number of values is limited. To demonstrate this, a sample size of $n=3$ was chosen. The other two values were arbitrarily chosen to be: $\delta=0.8, \eta=0.15$*.*

Let us set (without loss of generality) $h_{0}=0$. Since $h=\frac{1}{\delta}$ and therefore $h=1.25$, the considered values of $Y$ (using Eq. 6) are: $Y\in\left\{ -3.75,-2.5,-1.25,0,1.25,2.5,3.75 \right\}$

Using (Eq. 4), the probabilities of each value of $Y$ are depicted in Table 3.

Table 3 - Y distribution

| Value | Probability |
| --- | --- |
| -3.75 | $\Phi\left( \frac{-2.5\cdot1.25-0.15}{1} \right)-\Phi\left( \frac{-3.5\cdot1.25-0.15}{1} \right)\cong0.000526$ |
| -2.5 | $\Phi\left( \frac{-1.5\cdot1.25-0.15}{1} \right)-\Phi\left( \frac{-2.5\cdot1.25-0.15}{1} \right)\cong0.0209$ |
| -1.25 | $\Phi\left( \frac{-0.5\cdot1.25-0.15}{1} \right)-\Phi\left( \frac{-1.5\cdot1.25-0.15}{1} \right)\cong0.1977$ |
| 0 | $\Phi\left( \frac{0.5\cdot1.25-0.15}{1} \right)-\Phi\left( \frac{-0.5\cdot1.25-0.15}{1} \right)\cong0.4634$ |
| 1.25 | $\Phi\left( \frac{1.5\cdot1.25-0.15}{1} \right)-\Phi\left( \frac{0.5\cdot1.25-0.15}{1} \right)\cong0.2751$ |
| 2.5 | $\Phi\left( \frac{2.5\cdot1.25-0.15}{1} \right)-\Phi\left( \frac{1.5\cdot1.25-0.15}{1} \right)\cong0.0408$ |
| 3.75 | $\Phi\left( \frac{2.5\cdot1.25-0.15}{1} \right)-\Phi\left( \frac{1.5\cdot1.25-0.15}{1} \right)\cong0.0015$ |

Since the sample size is 3, there are 84 possible ways for these samples to spread over the 7 possible values. Starting with all 3 equal -3.75, followed by 2 equal -3.75 and one equals -2.5 and so on until all three equal 3.75. For each combination the probability (Eq. 11) and the average value must be calculated. To illustrate this step, the first 8 combinations are depicted in Table 4.

Table 4 - Combinations for n=3

| $Y$ values | | | | | | | $\bar{Y}$ | Probability |
| --- | --- | --- | --- | --- | --- | --- | --- | --- |
| -3.75 | -2.5 | -1.25 | 0 | 1.25 | 2.5 | 3.75 |
| 3 | 0 | 0 | 0 | 0 | 0 | 0 | -3.75 | 1.475E-10 |
| 2 | 1 | 0 | 0 | 0 | 0 | 0 | -3.33 | 1.75E-08 |
| 1 | 2 | 0 | 0 | 0 | 0 | 0 | -2.92 | 6.927E-07 |
| 2 | 0 | 1 | 0 | 0 | 0 | 0 | -2.92 | 1.656E-07 |
| 0 | 3 | 0 | 0 | 0 | 0 | 0 | -2.5 | 9.136E-06 |
| 1 | 1 | 1 | 0 | 0 | 0 | 0 | -2.5 | 1.31E-05 |
| 2 | 0 | 0 | 1 | 0 | 0 | 0 | -2.5 | 3.881E-07 |
| 0 | 2 | 1 | 0 | 0 | 0 | 0 | -2.08 | 0.0002593 |
| 76 more lines… | | | | | | | | … |
| Total | | | | | | | | 1.00 |

In the right column of Table 4 there are 19 possible values of $\bar{Y}$, ranging from -3.75 to 3.75. For each value the cumulative probability can be calculated, as depicted in Table 5.

Table 5 - $\bar{Y}$ distribution

| $\bar{Y}$ | $P(\bar{Y})$ |
| --- | --- |
| -3.75 | 1.47457E-10 |
| -3.33333 | 1.75048E-08 |
| -2.91667 | 8.5824E-07 |
| -2.5 | 2.26278E-05 |
| -2.08333 | 0.000352164 |
| -1.66667 | 0.003368534 |
| -1.25 | 0.020102063 |
| -0.83333 | 0.075137545 |
| -0.41667 | 0.176865788 |
| 0 | 0.262787019 |
| 0.416667 | 0.246276764 |
| 0.833333 | 0.145807035 |
| 1.25 | 0.054455678 |
| 1.666667 | 0.012770963 |
| 2.083333 | 0.001874032 |
| 2.5 | 0.000169559 |
| 2.916667 | 9.08671E-06 |
| 3.333333 | 2.6267E-07 |
| 3.75 | 3.14385E-09 |

Appendix 2 – Setting control limits

Based on the calculation of the $\bar{Y}$ distribution (Appendix 1, Table 3), the control limits can be calculated.

For this example, calculating from the table (according to Eq. 13) yields :

$$P\left(\overline{Y}>\frac{5}{3}\right)=0.002053>0.00135$$

For the next value we have :

$$P\left(\overline{Y}>\frac{25}{12}\right)=0.000179<0.00135$$

Then $UCL=\frac{5}{3}\cong$1.667

Similarly, since:

$P\left(\overline{Y}<-1.25\right)=0.00374>0.00135$ and $P\left(\overline{Y}<-\frac{5}{3}\right)=0.000376<0.00135$ then $LCL=-1.25$

Appendix 3 – Setting control limits

The discrete nature of the UCL and LCL functions (depicted in Figure 8) provides a useful way to create a simple table for the limits. Obviously, it is impractical to present the values for each case (i.e. combinations of $\delta, h, \sigma, \eta$ and the sample size - $n$). However, since the value of the UCL function 'jumps' at specific points and at constant 'stair' sizes, Table 6 provides a simple yet powerful way to obtain the required information. Each cell of the table contains ( first row) the value of the UCL for the case of $\eta=0$ (perfect symmetry). The following numbers are the 'jumping points' (i.e. the values of $\eta$ where the UCL increases). As the UCL always increases at specific values (always $\frac{h}{n}$), the required value of the UCL can be deduced. The procedure with an example is provided below.

Table 6 - UCL table

| *n* | Value of $\delta$ | | | | | |
| --- | --- | --- | --- | --- | --- | --- |
|  | 0.5 | 0.6 | 0.7 | 0.8 | 0.9 | 1 |
| 3 | 0.67  0.45 | 0.67  0.1 | 1  0.32 | 1  0.08; 0.5 | 1.33  0.26 | 1.33  0.07; 0.43 |
| 4 | 0.5  0.07 | 0.75  0.26 | 0.75  0.02; 0.38 | 1  0.15; 0.47 | 1.25  0.26 | 1.25  0.09; 0.36 |
| 5 | 0.6  0.29 | 0.6  0.05; 0.38 | 0.8  0.15; 0.43 | 1  0.22; 0.47 | 1  0.06; 0.28 | 1.2  0.13; 0.34 |
| 6 | 0.5  0.12; 0.45 | 0.67  0.2; 0.48 | 0.67  0.01; 0.25; 0.48 | 0.83  0.07; 0.28; 0.49 | 1  0.12; 0.31; 0.5 | 1.17  0.16; 0.33 |
| 7 | 0.43  0.01; 0.29 | 0.57  0.07; 0.31 | 0.71  0.12; 0.32 | 0.86  0.15; 0.33 | 0.86  0.01; 0.17; 0.33; 0.5 | 1  0.05; 0.2; 0.34; 0.49 |
| 8 | 0.5  0.17; 0.42 | 0.63  0.2; 0.41 | 0.63  0.03; 0.21; 0.39 | 0.75  0.06; 0.21; 0.37 | 0.88  0.08; 0.22; 0.36; 0.5 | 1  0.1; 0.23; 0.35; 0.48 |
| 9 | 0.44  0.09; 0.31 | 0.56  0.11; 0.3; 0.48 | 0.67  0.12; 0.28; 0.44 | 0.78  0.13; 0.27; 0.41 | 0.78  0.01; 0.13; 0.26; 0.38 | 0.89  0.03; 0.14; 0.25; 0.37; 0.48 |
| 10 | 0.4  0.03; 0.22; 0.42 | 0.5  0.05; 0.21; 0.38 | 0.6  0.06; 0.2; 0.34; 0.49 | 0.7  0.07; 0.19; 0.32; 0.44 | 0.8  0.07; 0.18; 0.29; 0.41 | 0.9  0.08; 0.18; 0.28; 0.38; 0.48 |
| 11 | 0.45  0.16; 0.33 | 0.55  0.15; 0.3; 0.45 | 0.55  0.01; 0.14; 0.27; 0.4 | 0.64  0.01; 0.13; 0.24; 0.36; 0.47 | 0.73  0.02; 0.12; 0.22; 0.32; 0.43 | 0.82  0.02; 0.12; 0.21; 0.3; 0.39; 0.49 |
| 12 | 0.42  0.1; 0.27; 0.43 | 0.5  0.09; 0.23; 0.37 | 0.58  0.09; 0.21; 0.32; 0.44 | 0.67  0.08; 0.18; 0.29; 0.39; 0.5 | 0.75  0.07; 0.17; 0.26; 0.35; 0.44 | 0.83  0.07; 0.15; 0.24; 0.32; 0.41; 0.49 |
| 13 | 0.38  0.06; 0.21; 0.36 | 0.46  0.05; 0.18; 0.31; 0.43 | 0.54  0.04; 0.15; 0.26; 0.37; 0.48 | 0.62  0.04; 0.13; 0.23; 0.33; 0.42 | 0.69  0.03; 0.12; 0.2; 0.29; 0.38; 0.46 | 0.77  0.03; 0.11; 0.18; 0.26; 0.34; 0.42; 0.5 |
| 14 | 0.36  0.02; 0.16; 0.3; 0.44 | 0.43  0.01; 0.13; 0.25; 0.37; 0.49 | 0.5  0.01; 0.11; 0.21; 0.32; 0.42 | 0.64  0.09; 0.18; 0.27; 0.36; 0.45 | 0.71  0.08; 0.16; 0.24; 0.32; 0.4; 0.48 | 0.79  0.07; 0.14; 0.21; 0.28; 0.36; 0.43; 0.5 |
| 15 | 0.4  0.12; 0.25; 0.38 | 0.47  0.1; 0.21; 0.32; 0.43 | 0.53  0.08; 0.17; 0.27; 0.36; 0.46 | 0.6  0.06; 0.14; 0.23; 0.31; 0.39; 0.48 | 0.67  0.05; 0.12; 0.2; 0.27; 0.34; 0.42; 0.49 | 0.73  0.04; 0.1; 0.17; 0.24; 0.31; 0.37; 0.44 |
| 16 | 0.38  0.09; 0.21; 0.33; 0.46 | 0.44  0.06; 0.17; 0.27; 0.38; 0.48 | 0.5  0.05; 0.14; 0.22; 0.31; 0.4; 0.49 | 0.56  0.03; 0.11; 0.19; 0.27; 0.34; 0.42; 0.5 | 0.63  0.02; 0.09; 0.16; 0.23; 0.3; 0.37; 0.44 | 0.69  0.01; 0.07; 0.14; 0.2; 0.26; 0.33; 0.39; 0.45 |
| 17 | 0.35  0.06; 0.17; 0.29; 0.41 | 0.41  0.04; 0.13; 0.23; 0.33; 0.43 | 0.47  0.02; 0.1; 0.19; 0.27; 0.36; 0.44 | 0.53  0.01; 0.08; 0.15; 0.23; 0.3; 0.37; 0.45 | 0.65  0.06; 0.13; 0.19; 0.26; 0.32; 0.39; 0.45 | 0.71  0.05; 0.1; 0.16; 0.22; 0.28; 0.34; 0.4; 0.46 |
| 18 | 0.33  0.03; 0.14; 0.25; 0.36; 0.47 | 0.39  0.01; 0.1; 0.2; 0.29; 0.38; 0.47 | 0.5  0.08; 0.16; 0.24; 0.31; 0.39; 0.47 | 0.56  0.05; 0.12; 0.19; 0.26; 0.33; 0.4; 0.47 | 0.61  0.04; 0.1; 0.16; 0.22; 0.28; 0.35; 0.41; 0.47 | 0.67  0.02; 0.08; 0.13; 0.19; 0.25; 0.3; 0.36; 0.42; 0.47 |
| 19 | 0.32  0.01; 0.11; 0.22; 0.32; 0.43 | 0.42  0.08; 0.17; 0.25; 0.34; 0.43 | 0.47  0.05; 0.13; 0.2; 0.28; 0.35; 0.43; 0.5 | 0.53  0.03; 0.1; 0.16; 0.23; 0.3; 0.36; 0.43; 0.49 | 0.58  0.02; 0.07; 0.13; 0.19; 0.25; 0.31; 0.37; 0.43; 0.49 | 0.68  0.06; 0.11; 0.16; 0.21; 0.27; 0.32; 0.37; 0.43; 0.48 |
| 20 | 0.35  0.09; 0.19; 0.29; 0.39; 0.48 | 0.4  0.06; 0.14; 0.22; 0.31; 0.39; 0.47 | 0.45  0.03; 0.1; 0.18; 0.25; 0.32; 0.39; 0.46 | 0.5  0.01; 0.08; 0.14; 0.2; 0.26; 0.33; 0.39; 0.45 | 0.6  0.05; 0.11; 0.16; 0.22; 0.28; 0.33; 0.39; 0.44; 0.5 | 0.65  0.03; 0.09; 0.14; 0.19; 0.24; 0.29; 0.34; 0.39; 0.44; 0.49 |

Step by Step Guide for using the UCL table

- Input: ${\mu,\sigma,h,h}_{0},n$ (example: a process that can be measured in multiplications of 10 (i.e. $h=10$). Mean 101.2, standard deviation of 8. The measuring steps ($h)$) .
- Step 1: Calculate parameters (in units of $\sigma$):

$h\leftarrow\frac{h}{\sigma}=\frac{10}{8}=1.25$

$\delta\leftarrow\frac{1}{h}=\frac{1}{1.25}=0.8$

| 1  0.08; 0.5 |
| --- |

$\eta\leftarrow\frac{\mu-h_{0}}{\sigma}=\frac{101.2-100}{8}=0.15$

- Step 2: Find the relevant cell ($n=3,\delta=0.8)$ in Table 6.
- Step 3: Find the value corresponding to the relevant $\eta$:
  - The value of the first line of the cell is 1.
  - There is a 'jump' at 0.08 and another one at 0.5.
  - Since the value of $\eta$ is 0.15, it lies after the first 'jump', but before the second.
  - Each 'jump' has the value of $\frac{1}{n}$.
  - Therefore, the value of UCL for all $0<\eta\leq0.5$ ; thusadd $\frac{1}{3}$.
  - The corresponding UCL is: $1+\frac{1}{3}=\frac{4}{3}$
- Step 4: Converting back to original units:

$UCL\leftarrow h\cdot UCL\cdot\sigma+h_{0}=1.25\times\frac{4}{3}\times8+100=113\frac{1}{3}$

Table 7 provides similar values for the LCL.

Table 7 - LCL table

| *n* | Value of $\delta$ | | | | | |
| --- | --- | --- | --- | --- | --- | --- |
|  | 0.5 | 0.6 | 0.7 | 0.8 | 0.9 | 1 |
| 3 | -0.67  0.27 | -0.67  0.48 | -1  0.16 | -1  0.35 | -1.33  0.13; 0.5 | -1.33  0.28 |
| 4 | -0.5  0.43 | -0.75  0.17 | -0.75  0.35 | -1  0.17; 0.48 | -1.25  0.03; 0.31 | -1.25  0.17; 0.42 |
| 5 | -0.6  0.12; 0.5 | -0.6  0.3 | -0.8  0.15; 0.43 | -1  0.04; 0.29 | -1  0.18; 0.4 | -1.2  0.09; 0.29; 0.49 |
| 6 | -0.5  0.22 | -0.67  0.09; 0.37 | -0.67  0.24; 0.48 | -0.83  0.15; 0.36 | -1  0.08; 0.26; 0.45 | -1.17  0.02; 0.19; 0.35 |
| 7 | -0.43  0.28 | -0.57  0.17; 0.41 | -0.71  0.1; 0.3; 0.5 | -0.86  0.04; 0.22; 0.4 | -0.86  0.15; 0.31; 0.47 | -1  0.1; 0.25; 0.39 |
| 8 | -0.5  0.08; 0.33 | -0.63  0.02; 0.23; 0.44 | -0.63  0.16; 0.34 | -0.75  0.11; 0.26; 0.42 | -0.88  0.07; 0.21; 0.35; 0.49 | -1  0.04; 0.16; 0.29; 0.42 |
| 9 | -0.44  0.14; 0.36 | -0.56  0.08; 0.27; 0.45 | -0.67  0.05; 0.2; 0.36 | -0.78  0.02; 0.16; 0.3; 0.44 | -0.78  0.12; 0.25; 0.37; 0.49 | -0.89  0.09; 0.21; 0.32; 0.43 |
| 10 | -0.4  0.18; 0.38 | -0.5  0.13; 0.3; 0.46 | -0.6  0.09; 0.24; 0.38 | -0.7  0.07; 0.19; 0.32; 0.44 | -0.8  0.05; 0.16; 0.27; 0.39; 0.5 | -0.9  0.04; 0.14; 0.24; 0.34; 0.44 |
| 11 | -0.45  0.03; 0.21; 0.39 | -0.55  0.01; 0.17; 0.32; 0.47 | -0.55  0.13; 0.26; 0.39 | -0.64  0.11; 0.22; 0.34; 0.45 | -0.73  0.09; 0.19; 0.29; 0.39; 0.5 | -0.82  0.08; 0.17; 0.26; 0.35; 0.44 |
| 12 | -0.42  0.07; 0.24; 0.4 | -0.5  0.05; 0.19; 0.33; 0.47 | -0.58  0.04; 0.16; 0.28; 0.4 | -0.67  0.04; 0.14; 0.24; 0.35; 0.45 | -0.75  0.03; 0.12; 0.22; 0.31; 0.4; 0.49 | -0.83  0.03; 0.11; 0.19; 0.28; 0.36; 0.44 |
| 13 | -0.38  0.1; 0.26; 0.41 | -0.46  0.09; 0.22; 0.34; 0.47 | -0.54  0.08; 0.19; 0.3; 0.41 | -0.62  0.07; 0.16; 0.26; 0.36; 0.45 | -0.69  0.06; 0.15; 0.23; 0.32; 0.41; 0.49 | -0.77  0.06; 0.14; 0.21; 0.29; 0.37; 0.44 |
| 14 | -0.36  0.13; 0.27; 0.42 | -0.43  0.11; 0.23; 0.35; 0.47 | -0.5  0.1; 0.2; 0.31; 0.41 | -0.64  0.01; 0.09; 0.18; 0.27; 0.36; 0.45 | -0.71  0.01; 0.09; 0.17; 0.25; 0.33; 0.41; 0.49 | -0.79  0.01; 0.09; 0.16; 0.23; 0.3; 0.37; 0.44 |
| 15 | -0.4  0.02; 0.15; 0.28; 0.42 | -0.47  0.03; 0.14; 0.25; 0.36; 0.47 | -0.53  0.03; 0.12; 0.22; 0.31; 0.41 | -0.6  0.03; 0.12; 0.2; 0.28; 0.37; 0.45 | -0.67  0.04; 0.11; 0.19; 0.26; 0.33; 0.41; 0.48 | -0.73  0.04; 0.11; 0.17; 0.24; 0.31; 0.38; 0.44 |
| 16 | -0.38  0.05; 0.17; 0.29; 0.42 | -0.44  0.05; 0.15; 0.26; 0.36; 0.47 | -0.5  0.05; 0.14; 0.23; 0.32; 0.41; 0.5 | -0.56  0.06; 0.14; 0.21; 0.29; 0.37; 0.45 | -0.63  0.06; 0.13; 0.2; 0.27; 0.34; 0.41; 0.48 | -0.69  0.06; 0.13; 0.19; 0.25; 0.31; 0.38; 0.44; 0.5 |
| 17 | -0.35  0.07; 0.19; 0.3; 0.42 | -0.41  0.07; 0.17; 0.27; 0.37; 0.46 | -0.47  0.07; 0.16; 0.24; 0.33; 0.41; 0.49 | -0.53  0.08; 0.15; 0.22; 0.3; 0.37; 0.44 | -0.65  0.01; 0.08; 0.15; 0.21; 0.28; 0.34; 0.41; 0.47 | -0.71  0.02; 0.08; 0.14; 0.2; 0.26; 0.32; 0.38; 0.44; 0.5 |
| 18 | -0.33  0.09; 0.2; 0.31; 0.42 | -0.39  0.09; 0.18; 0.28; 0.37; 0.46 | -0.5  0.01; 0.09; 0.17; 0.25; 0.33; 0.41; 0.49 | -0.56  0.02; 0.09; 0.16; 0.23; 0.3; 0.37; 0.44 | -0.61  0.04; 0.1; 0.16; 0.22; 0.28; 0.34; 0.41; 0.47 | -0.67  0.04; 0.1; 0.15; 0.21; 0.27; 0.32; 0.38; 0.43; 0.49 |
| 19 | -0.32  0.1; 0.21; 0.31; 0.42 | -0.42  0.02; 0.11; 0.19; 0.28; 0.37; 0.46 | -0.47  0.03; 0.11; 0.18; 0.26; 0.33; 0.41; 0.48 | -0.53  0.04; 0.11; 0.18; 0.24; 0.31; 0.37; 0.44; 0.5 | -0.58  0.05; 0.11; 0.17; 0.23; 0.29; 0.35; 0.4; 0.46 | -0.68  0.01; 0.06; 0.11; 0.17; 0.22; 0.27; 0.32; 0.38; 0.43; 0.48 |
| 20 | -0.35  0.02; 0.12; 0.22; 0.32; 0.42 | -0.4  0.04; 0.12; 0.2; 0.29; 0.37; 0.45 | -0.45  0.05; 0.12; 0.19; 0.26; 0.33; 0.41; 0.48 | -0.5  0.06; 0.12; 0.18; 0.25; 0.31; 0.37; 0.43; 0.5 | -0.6  0.01; 0.07; 0.12; 0.18; 0.24; 0.29; 0.35; 0.4; 0.46 | -0.65  0.03; 0.08; 0.13; 0.18; 0.23; 0.28; 0.33; 0.38; 0.43; 0.48 |
